# 5‐Fluorouracil (5‐FU) Cardiotoxicity: A Stressful Event

**DOI:** 10.1155/cric/2215209

**Published:** 2026-09-16

**Authors:** Yash Desai, Rohan Singh, Kush Desai, Samuel Bennett, Rohan Desai, Manu Mysore

**Affiliations:** ^1^ Department of Medicine, University of Maryland Medical Center, Baltimore, Maryland, USA, umm.edu; ^2^ Department of Biology, University of Maryland Baltimore County, Baltimore, Maryland, USA, umbc.edu; ^3^ Department of Cardiovascular Medicine, Department of Medicine, University of Maryland Medical Center, Baltimore, Maryland, USA, umm.edu

**Keywords:** 5-flurouracil, cardiotoxicity, heart failure

## Abstract

5‐Fluorouracil (5‐FU), a chemotherapeutic agent, is routinely used for solid tumors. Known cardiotoxic effects of 5‐FU include fatal arrhythmias, myocardial infarctions, and coronary vasospasms. This case report describes a 56‐year‐old man who had acute heart failure with cardiogenic shock suspected to be related to 5‐FU exposure; notably, cardiac dysfunction resolved following cessation of therapy.

**Keypoints**

• 5‐Fluorouracil (5‐FU), a chemotherapeutic agent that functions as a thymidylate synthase (TS) inhibitor, is used for treating gastric adenocarcinoma, pancreatic adenocarcinoma, breast adenocarcinoma, and colorectal adenocarcinoma. It is the second most common cause of chemotherapy‐induced cardiotoxicity [1].
• Cardiogenic shock is a rare complication of 5‐FU, and there is a lack of information regarding specific treatment options.
• The proposed mechanisms of 5‐FU–associated heart failure with cardiogenic shock include coronary vasospasm, direct myocardial injury, and vascular endothelial dysfunction [2].
• Acute heart failure associated with 5‐FU can be reversed by cessation of 5‐FU and by utilization of uridine triacetate.

## 1. Case Report

In this case report, we describe a case of suspected 5‐FU–associated cardiotoxicity presenting as acute heart failure with cardiogenic shock, with rapid and complete recovery following cessation of therapy.

## 2. Medical History

A 56‐year‐old male with no prior medical history was diagnosed with appendiceal cancer with peritoneal carcinomatosis. The patient underwent exploratory laparotomy with radical appendectomy, a diverting loop ileostomy, and hyperthermic intraperitoneal chemotherapy on June 9, 2023. The patient initiated Cycle 1 of combination chemotherapy with leucovorin calcium, 5‐FU, and oxaliplatin on August 9, 2023. Day 1 consisted of an infusion of oxaliplatin and leucovorin, followed by a bolus and continuous infusion of 5‐FU. Day 2 consisted of a leucovorin infusion, followed by a bolus and continuous infusion of 5‐FU.

## 3. Presentation and Physical Examination

The patient presented on Day 4 of Cycle 1 of chemotherapy with worsening shortness of breath and generalized weakness that began earlier that morning. He was completing a planned 48‐h 5‐FU continuous infusion at the time.

On exam, he appeared in moderate distress but was alert and oriented. He was hypotensive (MAP ~60 mmHg) and hypoxic, requiring nasal cannula oxygen supplementation, with dry mucous membranes and pale conjunctiva. Cardiovascular exam showed tachycardia with a regular rhythm and normal heart sounds. Pulmonary exam revealed respiratory distress with tachypnea and bilateral rales in the lower and middle lung fields. Abdominal exam was benign, and there was +1 pitting edema in both lower extremities.

## 4. Differential Diagnosis

• Cardiogenic shock due to myocardial ischemia versus drug‐induced cardiotoxicity versus myocarditis
• Septic shock secondary to pneumonia
• Pulmonary embolism

## 5. Management (Include Interventional, Surgical, and Medical, as Needed)

On admission, laboratory evaluation revealed elevated troponin levels peaking at 0.64 ng/mL, lactic acidosis with a maximum lactate of 3.7 mmol/L, a markedly elevated D‐dimer of 7177 ng/mL, an NT‐proBNP of 11,400 pg/mL, and leukocytosis with a white blood cell count of 21.5 K/cmm. Electrocardiogram showed sinus tachycardia with diffuse ST‐segment abnormalities.

Imaging studies supported signs of volume overload, and pulmonary embolism was ruled out. Given the differential of septic versus cardiogenic shock, the patient was empirically started on broad‐spectrum antibiotics, intravenous furosemide, and vasopressor/inotropic support with norepinephrine and dobutamine. Supplemental oxygen was provided via high‐flow nasal cannula.

A transthoracic echocardiogram revealed severe systolic dysfunction with an ejection fraction of 15%–20%, severe global left ventricular hypokinesis, and Grade I diastolic dysfunction. Cardiology consultation was obtained for new‐onset heart failure with suspected cardiogenic shock.

The patient was transitioned to a continuous furosemide infusion, resulting in a net negative fluid balance of 14 L over the course of hospitalization. This correlated with significant clinical improvement, including successful weaning off supplemental oxygen to room air and the discontinuation of dobutamine and norepinephrine by Hospital Day 3.

On Day 5, combined right and left heart catheterization was performed, revealing a right‐dominant coronary circulation without obstructive coronary artery disease. Hemodynamics were notable for low filling pressures: right atrial pressure of 1 mm Hg, right ventricular pressure of 18/1 mm Hg, pulmonary artery pressure of 13/5 mm Hg, and pulmonary capillary wedge pressure of 5 mm Hg, with a normal cardiac index of 2.7 L/min/m^2^ and cardiac output of 5.7 L/min.

Given the temporal relationship to chemotherapy, the oncology team was consulted for concern for possible 5‐FU–associated cardiotoxicity. The patient was treated with uridine triacetate 10 g orally every 6 h for a total of 20 doses as an antidote for suspected 5‐FU toxicity. As part of the oncologic workup, genetic testing for dihydropyrimidine dehydrogenase (DPD) deficiency revealed no pathogenic variants.

## 6. Outcome

The patient was discharged on Day 5 with planned cardio‐oncology follow‐up 2 weeks later. He completed the remaining doses of uridine triacetate at home. The patient underwent cardiovascular magnetic resonance imaging 2 weeks after discharge, which demonstrated resolution of heart failure with findings of normal left ventricular function and sized, no evidence of myocardial late gadolinium enhancement, mildly elevated myocardial extracellular volume fraction consistent with diffuse myocardial fibrosis, and right ventricular size and systolic function within normal limits.

The patient′s overall clinical course was most consistent with 5‐FU–associated stress cardiomyopathy. The patient and his oncologist were advised regarding cautious use of the agent moving forward.

## 7. Latest Follow‐Up

The patient later passed away after experiencing a pulmonary embolism. He was initiated on a heparin infusion for anticoagulation, but his course was complicated by gastrointestinal bleeding. After a joint discussion with his oncologist and family, the decision to transition to hospice care was made, shortly after which the patient passed away.

## 8. Discussion

Fluorouracil, also known as 5‐FU, was first established as a viable cancer treatment in 1957 [3]. Nowadays, it is widely used as a chemotherapeutic agent for various malignant solid tumors, including gastric, pancreatic, breast, and colorectal adenocarcinomas [4]. 5‐FU functions as an antimetabolite: After it enters cells, it inhibits the production of deoxythymidine monophosphate, a key component of DNA, and therefore inhibits cell growth and replication. In this way, it preferentially targets rapidly proliferating cells for destruction [4, 5]. Although 5‐FU is generally considered a well‐tolerated chemotherapy agent, side effects and toxicities can occur with administration, including fever, fatigue, mucositis, myelosuppression, GI distress, and neuropathy [4, 5]. Among the rarer but dangerous toxic effects of 5‐FU that have been documented is cardiotoxicity. In fact, 5‐FU is the second most common chemotherapy drug, second to anthracyclines, to cause cardiotoxicity [6].

There are numerous ways that 5‐FU–induced cardiotoxicity can manifest, such as angina, acute coronary syndrome, arrhythmias, myopericarditis, heart failure, and even death. The most common symptom that affected patients present with is chest pain, which can appear at rest or on exertion [6, 7]. In contrast, presentations dominated by acute left ventricular dysfunction and cardiogenic shock, as seen in this case, represent less frequent but more severe manifestations within the spectrum of fluoropyrimidine cardiotoxicity [8]. A review of 5‐FU cardiotoxicity [4] examined the literature from 1970 to 2017 and identified 37 studies meeting their search criteria [6]. The incidence of cardiotoxicity varied widely between the studies; however, some prospective studies found a 0% incidence [6, 7, 9], whereas one study examining capecitabine (a precursor drug to 5‐FU) found 34.6% of patients developed “cardiovascular symptoms” [10]. Notably, many of the studies were limited by a relatively low number of patients. Examining a couple of the larger studies that were included, Labianca et al. [11] was a retrospective study of 1083 patients that found an overall incidence of 1.6%, whereas a 1993 study by Keefe et al. [12] found that five out of 910 patients (0.55%) developed life‐threatening toxicity consistent with coronary artery vasospasm. According to Sara et al. [4], these differences in incidence can be attributed to differences in the drug being studied, the dose and method of administration, the use of concurrent chemotherapeutic agents with 5‐FU, patient characteristics, and the definition of cardiotoxicity used in the studies [4]. Regarding the dosing and method of administration, studies have consistently shown that 5‐FU cardiotoxicity most commonly occurs during the first cycle of administration, and more commonly occurs during continuous infusions as compared with bolus regimens [4, 5].

The two proposed mechanisms by which 5‐FU is thought to adversely affect the heart are ischemia and direct myocardial cell damage. Coronary vasospasm is the leading theory for the underlying mechanism of myocardial ischemia [4]. This phenomenon, which is brought on by either direct endothelial dysfunction or smooth muscle dysfunction, can produce EKG changes suggestive of vessel occlusion, including ST‐segment elevations, as well as troponin elevation. However, as was the case with our patient, such changes can occur in the absence of macrovascular coronary artery obstruction on coronary angiography. Regarding direct cellular damage, animal studies have demonstrated pathological changes to myocytes occurring in a dose‐dependent fashion. Evidence indicates that 5‐FU causes apoptosis of cardiomyocytes through oxidative stress and free radical release, but does not cause tissue necrosis [4, 5]. It is unclear what leads to the uncommon side effect of cardiomyopathy in patients who receive 5‐FU, but both human and animal autopsies implicate myocarditis as the causal factor [13]. According to Grunwald et al. [13], the tendency of 5‐FU to cause global hypokinesis lends more weight to myocarditis as a mechanism of cardiomyopathy as opposed to vascular causes, such as coronary spasm. These mechanisms are not mutually exclusive and may act in combination, which may help explain more severe presentations such as global systolic dysfunction and cardiogenic shock.

In this patient, suspected 5‐FU–associated cardiotoxicity was favored because symptoms began during active infusion, echocardiography showed new severe global rather than regional left ventricular dysfunction, coronary angiography showed no obstructive disease, and ventricular function subsequently normalized after withdrawal of therapy. Acute coronary syndrome therefore appeared less likely. Sepsis‐related cardiomyopathy was also considered, but the overall course and imaging favored a treatment‐related cardiac process. Myocarditis cannot be fully excluded, although the later cardiac MRI did not show late gadolinium enhancement, which provides some additional support against a persistent inflammatory or necrotic pattern.

While there are no standardized treatment options for 5‐FU cardiotoxicity, the pivotal first step in the management of 5‐FU–induced cardiotoxicity is immediate cessation of chemotherapy [5]. This can be followed by administration of antianginal agents such as calcium channel blockers or nitrates in patients with coronary spastic angina who developed myocardial ischemia on EKG during treatment [4, 5]. Moreover, uridine triacetate is an oral prodrug of uridine that has been approved by the Food and Drug Administration (FDA) as an antidote for overdose and serious acute adverse effects of 5‐FU or capecitabine [5, 14]. However, its role in cardiotoxicity is not well established, and available evidence is limited primarily to case reports [8, 15]. Following cessation of 5‐FU and administration of uridine triacetate, the patient demonstrated complete recovery of cardiac function; however, the relative contribution of each intervention cannot be definitely determined. Given the severity of presentation, future exposure to fluoropyrimidines in this patient would require careful multidisciplinary reassessment, as rechallenge after significant cardiotoxicity remains controversial [8].

The patient′s subsequent death from pulmonary embolism and bleeding complications highlights the broader systemic and prothrombotic milieu associated with advanced malignancy and likely represents an independent process from the acute cardiac presentation. While systemic illness and hypercoagulability could contribute to overall clinical instability, the temporal association with 5‐FU exposure and recovery of ventricular function after drug withdrawal support a primary treatment‐related cardiac process in this case.

Although causality cannot be definitively established in a single case report, the overall clinical course strongly supports suspected 5‐FU–associated cardiotoxicity. This case adds to prior reports showing that fluoropyrimidine cardiotoxicity can, in uncommon cases, present with fulminant but reversible left ventricular dysfunction and cardiogenic shock.

**Nomenclature**
5‐FU: 5‐fluorouracil

## Author Contributions

Yash Desai: manuscript writing and review, data collection, manuscript submission. Rohan Singh: manuscript writing, data collection. Kush Desai: manuscript writing. Samuel Bennett: manuscript writing. Rohan Desai: manuscript writing. Manu Mysore: conceptualization, manuscript review and editing, supervision.

## Funding

No funding was received for this manuscript.

## Conflicts of Interest

The authors declare no conflicts of interest.

## Data Availability

The data that support the findings of this study are available on request from the corresponding author. The data are not publicly available due to privacy or ethical restrictions.
